# The Clinical and Pathological Profile of BRCA1 Gene Methylated Breast Cancer Women: A Meta-Analysis

**DOI:** 10.3390/cancers13061391

**Published:** 2021-03-19

**Authors:** Ilary Ruscito, Maria Luisa Gasparri, Maria Paola De Marco, Flavia Costanzi, Aris Raad Besharat, Andrea Papadia, Thorsten Kuehn, Oreste Davide Gentilini, Filippo Bellati, Donatella Caserta

**Affiliations:** 1 Gynecology Division, Department of Medical and Surgical Sciences and Translational Medicine, Sant’Andrea University Hospital, Sapienza University of Rome, Via di Grottarossa 1035, 00189 Rome, Italy; demarco.mariapaola@gmail.com (M.P.D.M.); costanzi.flavia@gmail.com (F.C.); aris.besharat@gmail.com (A.R.B.); filippo.bellati@uniroma1.it (F.B.); donatella.caserta@uniroma1.it (D.C.); 2 Department of Gynecology and Obstetrics, Ente Ospedaliere Cantonale (EOC), Via Tesserete 46, 6900 Lugano, Switzerland; marialuisa.gasparri@eoc.ch (M.L.G.); andrea.papadia@eoc.ch (A.P.); 3 University of the Italian Switzerland (USI), Via Giuseppe Buffi 13, 6900 Lugano, Switzerland; 4 Interdisciplinary Breast Center, Department of Gynecology and Obstetrics, Klinikum Esslingen, 73730 Neckar, Germany; t.kuehn@klinikum-esslingen.de; 5 Breast Surgery Unit, San Raffaele University Hospital, via Olgettina 60, 20132 Milan, Italy; gentilini.oreste@hsr.it

**Keywords:** BRCA1, methylation, breast cancer, epigenetics, tumor progression, DNA

## Abstract

**Simple Summary:**

The aim of the present meta-analysis was to analyze all available studies reporting clinical characteristics of breast cancer gene 1 (BRCA1) gene hypermethylated breast cancer in women, and to pool the results in order to provide a unique clinical profile of this cancer setting population. Identifying the clinical profile of breast cancer in women harboring BRCA1 gene hypermethylation may help oncologists select a subgroup of patients who may be candidates for BRCA1 methylation assessment, thus, possibly enlarging the cancer population who may benefit from new target-therapy agents. Results showed that BRCA1 gene hypermethylation should be suspected in all breast cancer patients with advanced disease stages, positive lymph nodes, and premenopausal age at diagnosis. Multidisciplinary groups treating women with breast cancer should take into account the possibility of addressing patients with these characteristics with a BRCA1 gene methylation status analysis.

**Abstract:**

Background: DNA aberrant hypermethylation is the major cause of transcriptional silencing of the breast cancer gene 1 (BRCA1) gene in sporadic breast cancer patients. The aim of the present meta-analysis was to analyze all available studies reporting clinical characteristics of BRCA1 gene hypermethylated breast cancer in women, and to pool the results to provide a unique clinical profile of this cancer population. Methods: On September 2020, a systematic literature search was performed. Data were retrieved from PubMed, MEDLINE, and Scopus by searching the terms: “BRCA*” AND “methyl*” AND “breast”. All studies evaluating the association between BRCA1 methylation status and breast cancer patients’ clinicopathological features were considered for inclusion. Results: 465 studies were retrieved. Thirty (6.4%) studies including 3985 patients met all selection criteria. The pooled analysis data revealed a significant correlation between BRCA1 gene hypermethylation and advanced breast cancer disease stage (OR = 0.75: 95% CI: 0.58–0.97; *p* = 0.03, fixed effects model), lymph nodes involvement (OR = 1.22: 95% CI: 1.01–1.48; *p* = 0.04, fixed effects model), and pre-menopausal status (OR = 1.34: 95% CI: 1.08–1.66; *p* = 0.008, fixed effects model). No association could be found between BRCA1 hypermethylation and tumor histology (OR = 0.78: 95% CI: 0.59–1.03; *p* = 0.08, fixed effects model), tumor grading (OR = 0.78: 95% CI :0.46–1.32; *p* = 0.36, fixed effects model), and breast cancer molecular classification (OR = 1.59: 95% CI: 0.68–3.72; *p* = 0.29, random effects model). Conclusions: hypermethylation of the BRCA1 gene significantly correlates with advanced breast cancer disease, lymph nodes involvement, and pre-menopausal cancer onset.

## 1. Introduction

Breast cancer gene 1 (BRCA1) encodes a polyfunctional protein responsible for DNA repair, cell cycle control, protein ubiquitinoylation, and chromatin remodeling [1,2]. Germline mutations of BRCA1 are known to cause up to 45% of familial breast cancer, while germline aberrations in BRCA1 gene DNA sequences are involved in only 1% of sporadic breast cancer onsets [3]. Nevertheless, BRCA1 gene silencing was found to be a very frequent event in sporadic breast cancer, and it has been correlated with its progression and overall survival [4]. Methylation of CpG islands is an epigenetic mechanism involved in gene silencing. DNA aberrant hypermethylation was observed to be the major cause of transcriptional silencing of the BRCA1 gene, a phenomenon ranging from 13% to 40% in sporadic breast cancer [5,6].

While the clinicopathological characterization of germline BRCA1 mutated breast cancer has been well defined [7], there is still little known in regards to the pathological signature of BRCA1 gene hypermethylated breast cancer in women. During the last 15 years, several studies attempted to trace a clinical profile of these patients’ subset, but no unique results have been reported. Identifying the clinical features of BRCA1 gene hypermethylation in breast cancer patients is currently considered a major open question, since the definition of the clinical traits of this molecular signature could help oncologists to address methylated patients with dedicated treatment options and follow-up. In 2020, Kawachi et al. published the results of the first clinical trial reporting that human breast cancers with BRCA1 methylation showed a clinical response to PARP-inhibitors [8]. This is in line with what is already known in women with BRCA methylated ovarian cancer, who are currently suitable for treatment with Parp-Inhibitors even in front-line therapy [9]. This evidence has the potential to represent the paradigm shift of breast cancer treatment, by enlarging the cancer population who could benefit from these new targeted agents. In this scenario, the aim of the present meta-analysis was to analyze all available studies reporting clinical characteristics of BRCA1 gene hypermethylated breast cancer in women, and to pool the results in order to provide a unique clinical profile of this cancer setting population.

## 2. Materials and Methods

### 2.1. Data Identification and Selection

The present meta-analysis was carried out following the Preferred Reporting Items for Systematic reviews and Meta-Analyses (PRISMA) statement and included all studies without any restriction on publication year. On September 2020, a systematic literature search was performed. Data were retrieved from the electronic databases, PubMed, MEDLINE, and Scopus, by searching the terms: “BRCA*” AND “methyl*” AND “breast”. All English language original reports evaluating the association between BRCA1 gene methylation status and breast cancer patients’ clinicopathological features were considered for inclusion.

The reference list of original reports and reviews already published was also analyzed to identify other potential studies.

Review articles, case reports, editorials, and letters were excluded. Based on inclusion and exclusion criteria, two independent reviewers (IR and MLG) identified and selected the studies. Differences in the studies’ selection were resolved asking a third author (DC).

For each study included in the meta-analysis, the following data were recorded: first author’s information, publication year, study design, detection method, criteria to define BRCA1 gene hypermethylation for dichotomized methylation status (“hyper-” vs. “hypo-methylated”), sample size, percentage of BRCA1 gene hypermethylated cases, tumor histology, stage, grading, molecular classification, lymph nodal status, and patients’ menopausal status (pre- vs. postmenopausal women).

### 2.2. Endpoints

The primary endpoint was the association between BRCA1 gene methylation status and patients’ clinicopathological features, including tumor histology, stage, grading, lymph nodal status, tumor molecular classification, and patients’ menopausal status.

### 2.3. Statistical Analysis

The number of BRCA1 gene hypermethylated cases detected in association with each clinicopathological variable were stratified by studies, and the pooled odds ratio (OR) was calculated using a fixed- or a random-effects model. A χ2 test for heterogeneity among proportions was performed to assess the presence of statistical heterogeneity between studies. A fixed-effects model was applied in case statistical heterogeneity was not significant (I2 value ≤ 50%); differently, a random-effects model was used. Graphical representation of each study and pooled analysis were displayed by forest plots. The weight that each study provides in the meta-analysis was graphically reported as squares of different size. Confidence intervals (CIs) for each study were symbolized by the horizontal lines passing through the squares. The pooled OR was represented as a lozenge in the forest plot, and its size corresponded to the 95% CI of the OR. A *p* value ≤ 0.05 was considered significant.

Statistical analysis was performed using Review Manager 5.4 (http://www.cochrane.org (accessed on 16 March 2021)).

## 3. Results

**Figure 1:**
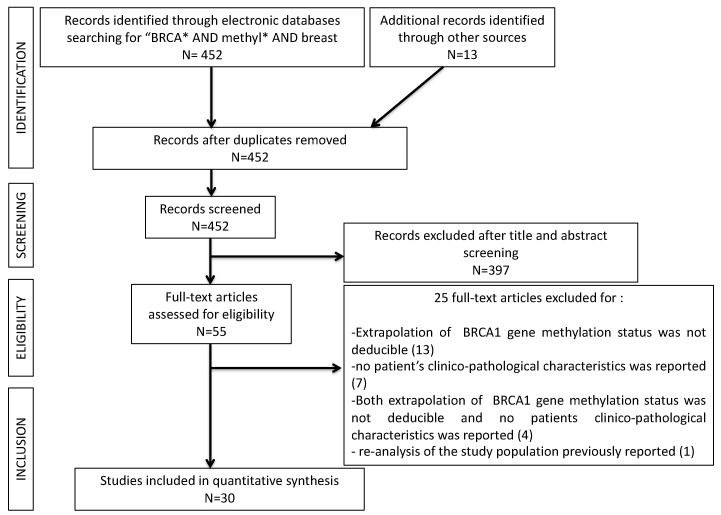
PRISMA flow-chart of the study selection process.

In total, 465 studies were retrieved through the literature search. Among these, 13 (2.8%) studies were removed as duplicates. A further 397 papers (85.4%) were excluded after title and abstract evaluation, being non-English-language original reports, studies not regarding breast cancers, studies performed on animals, review papers, or studies not evaluating the BRCA1 gene methylation status. Twenty-five (5.4%) studies were successively excluded after full-text evaluation: 13 were excluded because extrapolation of BRCA1 gene methylation status was not deducible [10,11,12,13,14,15,16,17,18,19,20,21,22]; seven other papers were excluded because no patient’s clinicopathological characteristics were reported [23,24,25,26,27,28,29]; four studies were eliminated for the coexistence of both the previous reasons [30,31,32,33]; one paper was excluded for the reanalysis of a study population that was previously reported [34]. Thirty (6.4%) studies remained for comparison at the end of the selection process. The PRISMA flow chart summarizing the process of evidence acquisition was shown in Figure 1. The flow chart maps out the number of studies identified, screened, included, and excluded, as well as the reasons for exclusions.

Globally, the total number of patients included in the meta-analysis was 3985, ranging from 26 to 851 patients per study. Thirteen [35,36,37,38,39,40,41,42,43,44,45,46,47], 12 [36,39,44,45,47,48,49,50,51,52,53,54], 10 [40,42,43,44,47,49,54,55,56,57], 18 [34,36,38,39,41,44,45,46,47,48,49,50,51,52,55,56,58,59], 9 [38,43,45,49,51,52,56,60,61], and 12 [34,35,38,45,47,49,50,51,52,58,62,63] studies reported data regarding the association between BRCA1 gene methylation status and breast cancer histology, disease stage, tumor grading, patients’ lymph nodal status, disease molecular classification, and patients’ menopausal status, respectively.

BRCA1 gene methylation status was evaluated by methylation-specific PCR (MS-PCR) in 23 studies [34,36,37,38,40,41,42,43,44,45,47,48,51,52,53,54,55,56,58,59,61,62,63]. The remaining seven studies adopted Southern Blots [35], PCR [57], MS-MLPA (Methylation-Specific Multiplex Ligation-dependent Probe Amplification) [39], Bisulfite sequencing PCR [50], combined bisulfite and restriction analysis (COBRA) [46], percentage of relative methylation (PRM) [60], and REMS-PCR (Restriction endonuclease-mediated selective PCR) [49] as a gene methylation detecting method, respectively. The main characteristics of the selected studies were listed in Table 1.

### 3.1. Correlation of BRCA1 Gene Methylation Status with Clinico-Pathological Variables

**Figure 2:**
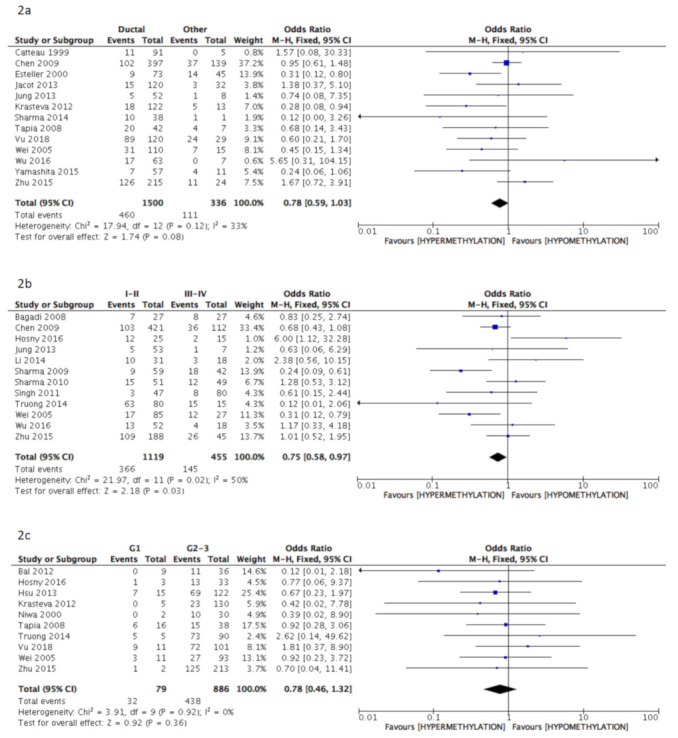

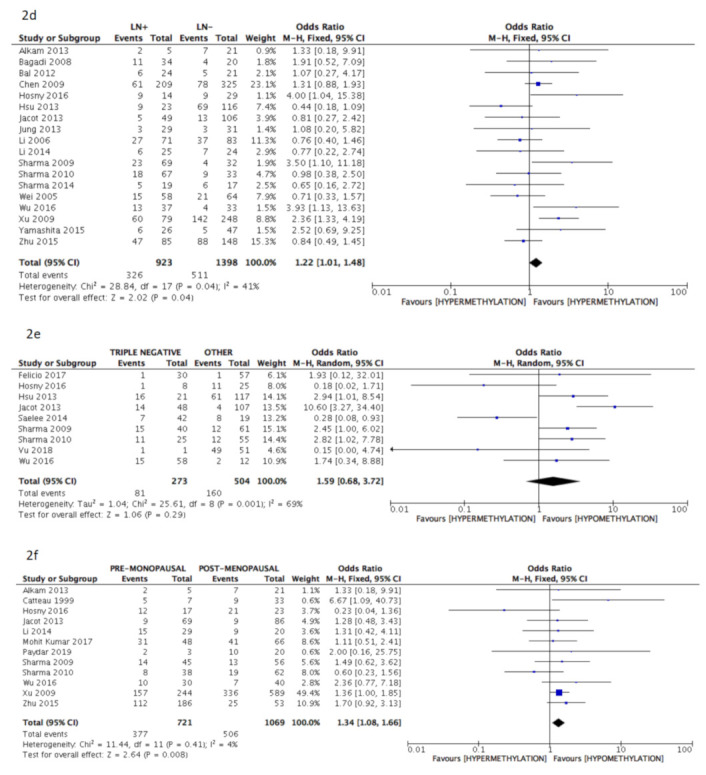
Pooled results on forest plots about the correlation between breast cancer gene 1 (BRCA1) gene methylation status with breast cancer patients’ clinicopathological characteristics. Events= number of BRCA1 gene hypermethylated cases. Figure shows the correlation between BRCA1 gene methylation and histology (2**a**), disease stage (2**b**), tumor grading (2**c**), lymph nodal status (2**d**), molecular classification (2**e**) and menopausal status (2**f**).

The pooled analysis data revealed a significant correlation between BRCA1 gene hypermethylation and advanced breast cancer disease stage, (OR = 0.75: 95% CI: 0.58–0.97; *p* = 0.03, fixed effects model, Figure 2b), lymph nodes involvement (OR = 1.22: 95% CI: 1.01–1.48; *p* = 0.04, fixed effects model, Figure 2d), and pre-menopausal status (OR = 1.34: 95% CI: 1.08–1.66; *p* = 0.008, fixed effects model, Figure 2f). On the contrary, no association could be found between BRCA1 gene hypermethylation and tumor histology (OR = 0.78: 95% CI: 0.59–1.03; *p* = 0.08, fixed effects model, Figure 2a), tumor grading (OR = 0.78: 95% CI: 0.46–1.32; *p* = 0.36, fixed effects model, Figure 2c), and breast cancer molecular classification (OR = 1.59: 95% CI: 0.68–3.72; *p* = 0.29, random effects model, Figure 2e).

#### Pooled Results of Studies Adopting Only the Methylation-Specific PCR (MS-PCR) Methodology for Detection of BRCA1 Methylation Status

As MS-PCR was the most adopted (by 23/30 included studies, 76.7%) methodology for the detection of BRCA1 methylation status, a subanalysis pooling only the results obtained by studies applying MS-PCR was carried out, in order to assess potential differences in results due to multiple methylation detection methods.

**Figure 3:**
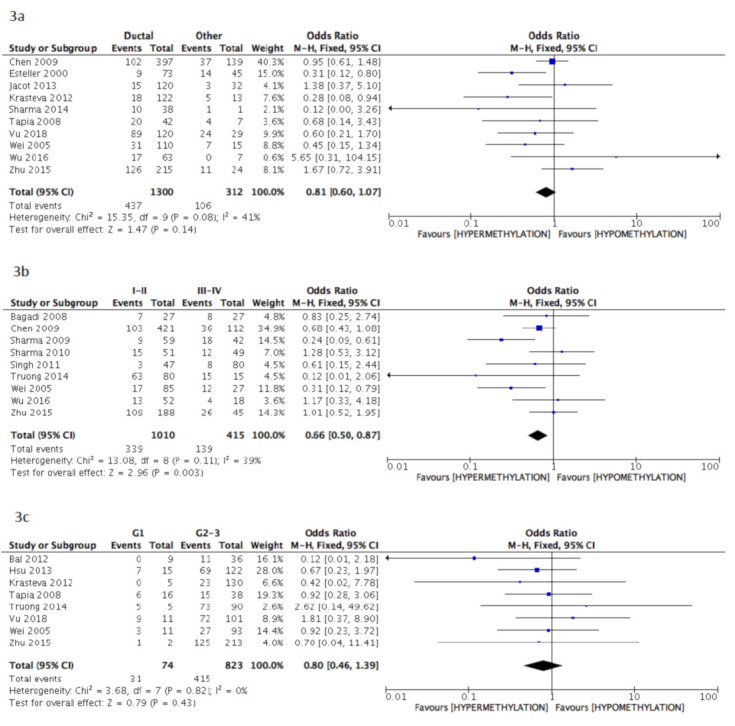

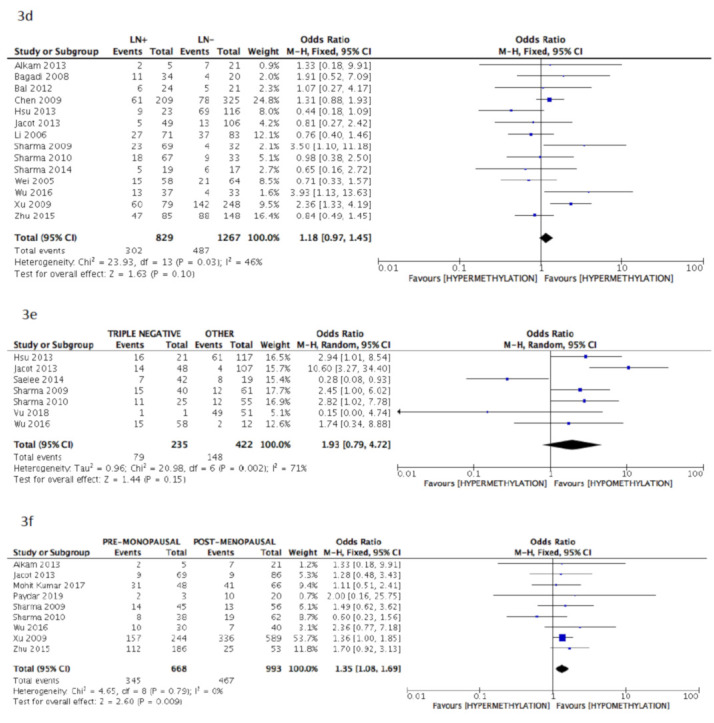
Pooled results on forest plots about the correlation between BRCA1 gene methylation status with breast cancer patients’ clinicopathological characteristics. In this subanalysis, only studies adopting methylation-specific PCR (MS-PCR) methodology for the detection of BRCA1 methylation status were included. Events= number of BRCA1 gene hypermethylated cases. Figure shows the correlation between BRCA1 gene methylation and histology (3**a**), disease stage (3**b**), tumor grading (3**c**), lymph nodal status (3**d**), molecular classification (3**e**) and menopausal status (3**f**).

The pooled data of the subanalysis confirmed a significant correlation between BRCA1 gene hypermethylation and advanced breast cancer disease stage, (OR = 0.66: 95% CI: 0.50–0.87; *p* = 0.003, fixed effects model, Figure 3b) as well as a positive correlation with patients’ pre-menopausal status (OR = 1.35: 95% CI: 1.08–1.69; *p* = 0.009, fixed effects model, Figure 3f), but lymph nodes involvement was not observed to be correlated with BRCA1 hypermethylation (OR = 1.18: 95% CI: 0.97–1.45; *p* = 0.10, fixed effects model, Figure 3d). Additionally, in this case, no association was found between BRCA1 gene hypermethylation and tumor histology (OR = 0.81: 95% CI: 0.60–1.07; *p* = 0.14, fixed effects model, Figure 3a), tumor grading (OR = 0.80: 95% CI: 0.46–1.39; *p* = 0.43, fixed effects model, Figure 3c), and breast cancer molecular classification (OR = 1.93: 95% CI: 0.79–4.72; *p* = 0.15, random effects model, Figure 3e).

## 4. Discussion

“Molecular tumor boards” have increasing become a reality worldwide for referral multidisciplinary oncologic centers, aiming to personalize cancer treatments for each single patient [64]. In this context, breast cancer has been a pioneering model for integrating surgery, oncology, histology, and genetics with molecular biology, with “Breast Units” being the most successful example of an integrated approach to cancer patients, under the leading principle “from the bench to the bedside”.

So far, huge advances have been carried out in the clinical application of molecular biology, and we are now facing the challenging step of going over cancer biology and cancer genetics by considering epigenetics as an essential part of cancer diagnostics and therapy [65].

Epigenetics groups post-transcriptional modifications of the genetic information undertaken basically through the DNA methylation phenomenon. DNA methylation refers to the addition of a methyl group (CH3) to the cytosine residue of a cytosine–guanidine pair, a CpG dinucleotide, in the DNA sequence. DNA methylation is a pivotal mechanism in early development, the so called “epigenetic reprogramming” event [66]. In adult cells, DNA methylation has been extensively demonstrated to be involved in the onset and progression of cancer, mainly through the silencing of tumor suppressor genes such as BRCA1, ATM, and PALB2 [67,68,69].

Up to now, in breast cancer patients, the investigation of BRCA1/2 gene epigenetic silencing has not routinely been included into the clinical algorithm of patients’ profiling and therapeutic approach. This currently limits the patients’ access to a wider platform of biological treatment, such as parp-inhibitors [8].

Identifying the clinical profile of breast cancer women harboring BRCA1 gene hypermethylation may help oncologists to select the subgroup of patients who may be candidates for BRCA1 methylation assessment, thus, possibly enlarging the cancer population who may benefit from new target-therapy agents.

This meta-analysis showed that BRCA1 gene hypermethylation in breast cancer significantly correlated with advanced disease stage, lymph nodal involvement, and pre-menopausal age at diagnosis. On the contrary, triple negative molecular classification, as well as advanced tumor grading and ductal histology, were not found to be more represented in the pooled group of BRCA1 hypermethylated breast cancer patients.

To our knowledge, the present meta-analysis is the first study which systematically investigates the role of BRCA1 gene hypermethylation in breast cancer patients’ clinicopathological characteristics. Involving 3985 patients, our results can be considered more reliable than those reported in each of single included studies. Nevertheless, our findings may be accompanied by some limitations. First, the definition of “hypermethylated” BRCA1 gene was heterogeneous among studies.

Second, the techniques applied for the detection of BRCA1 methylation status included seven different methodologies (MS-PCR, Southern Blots, PCR, MS-MLPA, Bisulfite sequencing PCR, combined bisulfite and restriction analysis (COBRA), percentage of relative methylation (PRM), and REMS-PCR). As the global debate concerning the attempt to identify a unique technique for the detection BRCA1 methylation status still ongoing, the authors decided to include in the present meta-analysis the studies carried out with all currently adopted technologies, each considering different definitions of a hypermethylated BRCA1 gene. Nevertheless, a subanalysis pooling only the studies adopting MS-PCR, as the methodology applied in 77% of included studies, was carried out, substantially confirming the result of the global pooled analysis. Until the international scientific community defines the most appropriate methodology for the assessment of BRCA methylation status, which will be accompanied by a unique laboratory definition of hypermethylated BRCA, it will not be possible to draw definitive conclusions on the clinical profile of this molecular signature.

Third, the study design of the included reports was retrospective in the vast majority of cases. Prospective studies on BRCA1 gene methylation status on a large cohort of breast cancer women was strongly awaited.

## 5. Conclusions

The present meta-analysis showed that BRCA1 gene hypermethylation should be suspected in all breast cancer patients with advanced disease stages, positive lymph nodes, and premenopausal age at diagnosis. Multidisciplinary groups treating women with breast cancer should take into account the possibility of addressing patients with these characteristics with a BRCA1 gene methylation status analysis.

No association between BRCA1 methylation status and other clinicopathological variables (such as tumor histology, tumor grading, and tumor molecular classification) was identified.

Larger sample-size prospective studies and a unanimous methodology to determine BRCA gene methylation status, as well as a unique laboratory definition of BRCA1 hypermethylated cases, will help in future to draw definitive conclusions about the clinical signature of BRCA1 gene methylated breast cancer in women.

## Figures and Tables

**Table 1:** Characteristics of the included studies.

References	Year of Publication	Study Type	Method	BRCA Hypermethylation Definition	Pts Number	Methylated BRCA Cases (%)
Catteau A et al [35]	1999	Experimental study	Southern blots	Presence of an additional 3765 bp fragment of both SmaI sites in the BRCA1 promoter region.	96	11 (11.4%)
Manel Esteller et al [37]	2000	Experimental study	MS-PCR	changes produced following bisulfite treatment of DNA, which converts unmethylated, but not methylated	118	23 (19.4%)
Youko Niwa et al [57]	2000	Experimental study	PCR	observing whether the band specific to the BRCA1 gene after digestion of breast cancer DNA with Hha I restriction enzyme.	32	10 (31%)
Wei M et al [44]	2005	Retrosp. cohort	MS-PCR	Presence of specific primer sequences of BRCA1 reaction for the methylated	131	39 (29.8%)
Li S Y et al [59]	2006	Retrosp. cohort	MS-PCR	Specific primer sequences of BRCA1 for the methylated reaction. Negative (blood DNA) and positive (colorectal or breast cancer DNA) controls as well as a blank (no DNA) were run with each PCR assay.	193	80 (41%)
Tapia T et al [42]	2008	Retrosp. cohort	MS-PCR	Presence of specific primer sequences of BRCA1 reaction for the methylated	49	24 (48.9%)
Bagadi R et al [45]	2008	Retrosp. cohort	MS-PCR	Presence of specific primer sequences of BRCA1 reaction for the methylated	54	15 (28%)
Xu X et al [32]	2009	Retrosp. cohort	MS-PCR	Presence of specific primer sequences of BRCA1 reaction for the methylated	851	504 (59.2%)
Chen et al [34]	2009	Retrosp. cohort	MS-PCR	Presence of specific primer sequences of BRCA1 reaction for the methylated	536	139 (25.9%)
Sharma et al [52]	2009	Retrosp. cohort	MS-PCR	Two sets of primers were designed for each gene, one specific for DNA methylated at the promoter region and the other specific for unmethylated DNA.	100	27 (27%)
Sharma et al [51]	2010	Retrosp. cohort	MS-PCR	Presence of specific primer sequences of BRCA1 reaction for the methylated	100	27 (27%)
Singh A K et al [53]	2011	Retrosp. cohort	MS-PCR	sequence changes produced, following the bisulfite treatment of DNA, which converts unmethylated, but not methylated, cytosines to uracil.	127	11 (8.7%)
Bal A. et al [55]	2012	Retrosp. cohort	MS-PCR	Presence of specific primer sequences of BRCA1 reaction for the methylated	45	11 (24 %)
Krasteva M. E et al [40]	2012	Retrosp. cohort	MS-PCR	Presence of specific primer sequences of BRCA1 reaction for the methylated	135	23 (17%)
Jung EU et al [39]	2013	Retrosp. cohort	MS-MLPA	if the target DNA is methylated, the hemi-methylated probe/sample DNA hybrids are prevented from digestion by HhaI and the target region is amplified, generating a signal.	60	6 (10%)
Jacot W et al [38]	2013	Retrosp. cohort	MS-PCR	Presence of specific primer sequences of BRCA1 reaction for the methylated	155	18(11.7%)
Alkam J et al 2013 [58]	2013	Retrosp. cohort	MS-PCR	Presence of specific primer sequences of BRCA1 reaction for the methylated	26	9 (35%)
Hsu NC et al 2013 [56]	2013	Retrosp. cohort	MS-PCR	Presence of specific primer sequences of BRCA1 reaction for the methylated	139	78 (56%)
Saelee P et al [61]	2014	Retrosp. cohort	MS-PCR	Presence of specific primer sequences of BRCA1 reaction for the methylated	61	15 (24.6%)
Truong PK et al [54]	2014	Retrosp. cohort	MS-PCR	Presence of specific primer sequences of BRCA1 reaction for the methylated	95	78 (82.1%)
Sharma et al [41]	2014	Retrosp. cohort	MS-PCR	Presence of specific primer sequences of BRCA1 reaction for the methylated	37	11 (30%)
Li Q et al [50]	2014	Retrosp. cohort	bisulfite sequencing PCR	Five positive clones for each sample were selected and analyzed using the ABI 3730 DNA Sequencer (Applied Biosystems). The percentage of methylation for each sample was calculated as the number of methylated CpG dinucleotides /(5 × 48) × 100%.	49	24 (49%)
Yamashita N et al [44]	2015	Retrosp. cohort	combined bisulfite and restriction analyses (COBRA)	The PCR fragment contains two Hha I recognition sites, which are differentially digested when the template DNA is methylated at each respective site	69	11 (16%)
Zhu X et al [45]	2015	Retrosp. cohort	MS-PCR	Presence of specific primer sequences of BRCA1 reaction for the methylated	239	137 (57.3 %)
Hosny MM et al [49]	2016	Retrosp. cohort	REMS-PCR	The presence of bands with sizes of 500 bp indicated methylation of BRCA1	40	17 (42.5%)
Wu L et al [45]	2016	Retrosp. cohort	MS-PCR	Presence of specific primer sequences of BRCA1 reaction for the methylated	70	17 (24.3%)
Felicio PS et al [60]	2017	Retrosp. cohort	Percentage of Relative Methylation (PRM)	to classify the samples as methylated or unmethylated, a cut-off of 4% was set	88	2 (2,3%)
Mohit Kumar M et al [62]	2017	Retrosp. cohort	MS-PCR	Presence of specific primer sequences of BRCA1 reaction for the methylated	114	55 (48.2%)
Vu LT et al [43]	2018	Retrosp. cohort	MS-PCR	Presence of specific primer sequences of BRCA1 reaction for the methylated	149	113 (58.23 %)
Paydar P et al [63]	2019	Retrosp. cohort	MS-PCR	Presence of specific primer sequences of BRCA1 reaction for the methylated	27	12 (44.4%)
